# Amplification-Driven S100A11 Overexpression in Hepatocellular Carcinoma Is Associated with Metabolic Reprogramming, ECM Remodelling, and Immune Evasion: A Pan-Cancer Genomic Study

**DOI:** 10.3390/cancers18172848

**Published:** 2026-09-03

**Authors:** Stuart Lutimba, Eiman Aleem

**Affiliations:** Cancer, Infection and Therapeutics Research Group, School of Allied Health and Life Sciences, College of Health and Life Sciences, London South Bank University, 103 Borough Road, London SE1 0AA, UK; stuart.lutimba@lsbu.ac.uk

**Keywords:** S100A11, pan-cancer, hepatocellular carcinoma, overexpression, amplification, metabolic reprogramming, ECM, immune evasion

## Abstract

Background: S100A11, a calcium-binding S100 family protein, is increasingly implicated in carcinogenesis, yet its molecular regulation and clinical relevance across cancers remain unclear. Hepatocellular carcinoma (HCC) carries a dismal prognosis, in part due to a lack of reliable biomarkers for risk stratification of established disease. Methods: We conducted a pan-cancer analysis of S100A11 genomic alterations across 31 studies (10,767 samples) obtained from TCGA, encompassing copy number alterations, somatic mutations, and DNA methylation. HCC-specific analyses evaluated S100A11 expression, its potential as a diagnostic/prognostic marker, co-expression networks, and pathway enrichment using TCGA-LIHC data, with univariate and multivariate Cox regression to assess survival associations. Results: S100A11 alterations were predominantly driven by copy number amplification, with the highest frequencies in hepatobiliary cancers, lung and breast cancers. Copy number amplification showed a consistent inverse relationship with promoter methylation, indicating amplification-driven transcriptional activation. In HCC, S100A11 was markedly overexpressed compared with normal liver tissue, with strong diagnostic discriminatory capacity. High S100A11 expression was significantly associated with inferior overall survival (log-rank *p* = 0.032; HR = 1.46, 95% CI 1.03–2.06) and remained an independent predictor of overall survival after adjustment for age, sex, and AJCC pathologic stage (HR = 1.27, 95% CI 1.01–1.60, *p* = 0.038). Co-expression and pathway analyses demonstrated an association between S100A11 and metabolic reprogramming, extracellular matrix remodelling, and immune dysregulation. Conclusions: These findings identify S100A11 as a candidate diagnostic and prognostic biomarker in HCC whose overexpression is associated with metabolic reprogramming, ECM remodelling, and immune dysregulation, warranting experimental validation of a mechanistic role.

## Simple Summary

S100A11, a calcium-binding S100 family protein, is increasingly implicated in carcinogenesis, yet its molecular regulation and clinical relevance across cancers remain unclear. Hepatocellular carcinoma (HCC) has poor prognosis, in part due to less effective patient stratification. In the present study, we integrated genomic, epigenetic, and transcriptomic data to characterise S100A11 alterations in HCC and assess their association with patient survival, with the aim of defining its potential as a diagnostic and prognostic biomarker. Increase in S100A11 copy number emerged as the dominant mechanism of dysregulation, inversely associated with methylation changes in the gene. Analysis of co-expressed genes associated S100A11 with metabolic reprogramming, extracellular matrix remodelling, and an immunosuppressive tumour microenvironment, while higher S100A11 expression was significantly associated with poor survival and correlated with more advanced tumour stage. These findings identify S100A11 as a candidate prognostic marker in HCC and suggest new avenues for understanding its contribution to disease progression.

## 1. Introduction

S100A11 is a member of the S100 family of small calcium-binding proteins, which play key roles in calcium-dependent signal transduction. Members of this family regulate diverse cellular processes, including cytoskeletal organisation, cell cycle progression, differentiation, migration, and stress responses, many of which are frequently dysregulated in cancer [1]. The S100 protein family comprises 25 proteins widely expressed in various cell types and tissues [2]. S100A11 has been implicated in membrane repair, actin remodelling, and transcriptional regulation, highlighting its potential role in tumourigenesis [3]. Emerging evidence has also highlighted a role for S100A11 in regulating focal adhesion dynamics [4].

Increasing evidence suggests that S100A11 is aberrantly expressed in a wide range of malignancies and contributes to cancer progression in a context-dependent manner. Elevated S100A11 expression has been reported in pancreatic, colorectal, breast, and liver cancers, where it has been associated with enhanced proliferation, epithelial–mesenchymal transition (EMT), invasion, and metastatic capacity [3,5,6,7,8]. Mechanistically, S100A11 has been shown to modulate oncogenic pathways such as TGF-β, PI3K/AKT, and MAPK signalling, thereby promoting tumour aggressiveness and resistance to therapy [6,8]. However, despite these findings, the extent to which S100A11 acts as a shared oncogenic driver versus its cancer-specific function remains unclear.

Pan-cancer genomic and transcriptomic analyses provide a powerful framework to address this question by enabling systematic comparison of genomic alterations and gene expression patterns across multiple cancer types. Large-scale datasets generated by The Cancer Genome Atlas (TCGA) have revealed both common and cancer-specific features, improving our understanding of tumour heterogeneity and identifying novel therapeutic vulnerabilities [9]. Applying a pan-cancer approach to S100A11 allows for evaluation of its genomic variations and transcriptional dysregulation across different types of cancer, its association with key oncogenic pathways, and its potential prognostic relevance.

Our pan-cancer analysis of S100A11 genetic alterations identified hepatocellular carcinoma (HCC), breast invasive carcinoma, lung adenocarcinoma (LAC), and cholangiocarcinoma as conditions exhibiting the highest frequencies of copy number alterations. The ranking of both hepatobiliary cancers in the top four of the cohort suggested the liver as a site of recurrent S100A11 genomic activation and provided the rationale for the subsequently focused detailed transcriptional analysis of S100A11 in HCC.

HCC is the most common primary liver cancer and represents a major global health burden, ranking among the leading causes of cancer-related mortality worldwide [10]. Despite advances in targeted therapies and immunotherapy, clinical outcomes for patients with advanced HCC remain poor, largely due to extensive molecular heterogeneity and limited biomarkers for patient stratification, as well as relatively late detection of the disease. Comprehensive genomic and transcriptomic profiling has demonstrated that HCC is characterised by recurrent alterations in pathways regulating cell cycle control, WNT/β-catenin signalling, telomere maintenance, oxidative stress, and epigenetic regulation (reviewed in [11,12]). Transcriptome-based classifications have further identified biologically distinct HCC subtypes associated with proliferation, immune activity, and metabolic reprogramming, each with prognostic and therapeutic implications [13]. Within this complex molecular landscape, dysregulation of calcium-binding proteins and stress-response pathways has emerged as an important contributor to hepatocarcinogenesis.

Notably, increased S100A11 expression has been reported in HCC tissues compared with adjacent non-tumour liver and has been associated with poor differentiation, vascular invasion, and unfavourable clinical outcomes [8,14]. Furthermore, in a study comparing the functions of the closely related S100A10 and S100A11, S100A11 knockdown consistently reduced metabolic dysfunction-associated steatotic liver disease (MASLD) and tumoral growth in complementary mouse models of MASLD and HCC. It also triggered concomitant partial loss of endogenous protective S100A10 [15]. However, existing studies are largely limited to functional models or individual cohorts, and a comprehensive transcriptomic characterisation of S100A11 in HCC within a broader pan-cancer context is limited. A focused analysis of S100A11 expression and its molecular associations in HCC is therefore critically needed to clarify its biological relevance, identify co-expressed gene networks and pathways, and assess its potential as a prognostic biomarker or therapeutic target.

In this study, we conducted a pan-cancer genomic analysis of S100A11 using publicly available datasets and focused our transcriptional analysis on HCC. By integrating genomic alterations, differential expression, survival analyses, and pathway enrichment approaches, we aimed to elucidate the cancer-specific features associated with S100A11 and to define its potential prognostic and diagnostic role in HCC.

## 2. Materials and Methods

### 2.1. Multi-Cohort Genomic Alteration Profiling of S100A11

S100A11 genomic alterations were profiled across the TCGA PanCancer Atlas collection on cBioPortal (31 publicly available studies, 10,767 samples; accessed via the cBioPortalData Bioconductor package, (https://www.cbioportal.org/), [accessed on 10 December 2025]). The study assessed multiple types of genomic alterations, including copy number variations (amplifications, gains, shallow deletions, and deep deletions) and somatic point mutations from TCGA (https://www.cancer.gov/ccg/research/genome-sequencing/tcga, [accessed on 10 December 2025]).

DNA methylation, mutational signatures and clonality associated with S100A11 were comprehensively analysed using TCGA datasets. Promoter methylation levels at the cg12052258 probe were extracted from Illumina HM27 and HM450 platforms and quantified as β-values, with samples stratified by copy number and mutation status to evaluate epigenetic regulation in relation to genomic alterations. DNA methylation levels are quantified as β-values derived from methylation array intensity measurements, where β represents the ratio of methylated to total signal (methylated + unmethylated). β-values range from 0.0 to 1.0, with β < 0.2 conventionally defined as hypomethylated, 0.2–0.8 as intermediate methylation, and β > 0.8 as hypermethylated. This normalisation approach enables direct comparison of methylation status across samples and platforms.

Mutational processes were assessed using doublet base substitution signature contribution scores, while variant allele frequency (VAF) was used to infer clonality of S100A11 missense mutations and loss of heterozygosity in diploid samples.

### 2.2. Transcriptomic Analysis of S100A11 in the TCGA-LIHC Cohort

RNA sequencing data and corresponding clinical information for HCC patients were obtained from TCGA Liver Hepatocellular Carcinoma (TCGA-LIHC) project through the Genomic Data Commons (GDC) Data Portal (https://portal.gdc.cancer.gov/, [accessed on 10 December 2025]). The dataset comprised 371 primary tumour specimens and 50 matched normal liver tissues. Gene expression data were generated using the STAR (Spliced Transcripts Alignment to a Reference) workflow, with raw counts normalised and downloaded via the TCGAbiolinks R package (version 2.28.0).

Raw RNA-seq count data were log2-transformed after adding a pseudocount of 1 to avoid undefined logarithmic values [log2(counts + 1)]. Samples were classified as tumour or normal tissue based on TCGA sample type definitions. For survival analyses, patients were dichotomized into high and low S100A11 expression groups using the median expression value as the cutoff, a standard approach in biomarker studies to ensure balanced group sizes. Multivariate Cox regression adjusted for age at diagnosis, sex, and the American Joint Committee on Cancer (AJCC) pathologic stage (Stage I–IV, collapsed from AJCC substages) was conducted. Because AJCC stage was not recorded for all patients, the stage-adjusted model was restricted to the complete-case subset (n = 239 of 304 patients with survival data); this complete-case, stage-adjusted model is the single multivariable model reported in Section 3.5.

### 2.3. Co-Expression Network and Functional Enrichment Analysis of S100A11-Associated Genes in Hepatocellular Carcinoma

Differentially expressed genes (DEGs) associated with S100A11 were identified by comparing S100A11-high versus S100A11-low HCC tumour samples using the limma R package (version 3.56.2). RNA-seq counts were normalised via voom transformation, followed by linear modelling and empirical Bayes moderation. DEGs were defined by false-discovery-rate (FDR)-adjusted *p*-value < 0.05 and log2 fold change > 1. The top 50 DEGs (ranked by adjusted *p*-value) were visualised in a heatmap in Section 3.7.

Co-expression analysis involved computing Pearson correlation coefficients between S100A11 and all other genes in tumour samples. The top 50 genes with strongest correlation (|r| > 0.5, *p* < 0.001) were selected for functional interrogation. Hierarchical clustering (Pearson correlation distance, complete linkage) was applied to delineate co-expression modules.

Gene Ontology (GO) and Kyoto Encyclopaedia of Genes and Genomes (KEGG) pathway enrichment analyses were performed using clusterProfiler (version 4.8.2), with gene symbol-to-Entrez ID mapping via org.Hs.eg.db. Significantly enriched terms (adjusted *p* < 0.05, Benjamini–Hochberg) were ranked by gene ratio; the top 15 were visualised.

### 2.4. Evaluation of S100A11 as a Prognostic and Diagnostic Biomarker

Overall survival (OS) was assessed using Kaplan–Meier curves generated with the survival R package (version 3.8.3), stratifying patients by S100A11 expression (high vs. low; median cutoff) and comparing groups via log-rank test (*p* < 0.05). Number-at-risk tables were included.

Prognostic impact of S100A11 genomic alterations (altered n = 399 vs. unaltered n = 10,140, total n = 10,539) was examined using Kaplan–Meier curves and log-rank tests. Univariate and multivariate Cox proportional hazards models evaluated S100A11 expression (continuous, log2-transformed) as an independent prognostic factor, adjusting for age, sex, and AJCC pathologic stage (Stage I-IV) in HCC. Proportional hazards assumption was confirmed via Schoenfeld residuals. Hazard ratios (95% CI) were visualised in forest plots.

### 2.5. Data Analysis and Visualisation

All analyses were conducted in R (version 4.3.2). Visualisations employed ggplot2 (version 3.5.2), ComplexHeatmap (version 2.18.0), pheatmap (version 1.0.13), survminer (version 0.5.1), and cowplot (version 1.2.0) packages. Heatmaps displayed row Z-score-normalised expression with clinical annotations. Genomic alteration plots (OncoPrint, copy number, methylation) used standardised colour schemes and transparency for clarity.

Statistical rigour included two-sided tests, FDR correction, complete-case analysis for missing data, and explicit reporting of sample sizes. Non-parametric (Spearman) and categorical (Fisher’s exact) tests were applied as appropriate.

## 3. Results

### 3.1. Pan-Cancer Genomic Landscape and Tissue-Specific Alterations of S100A11

**Figure 1:**
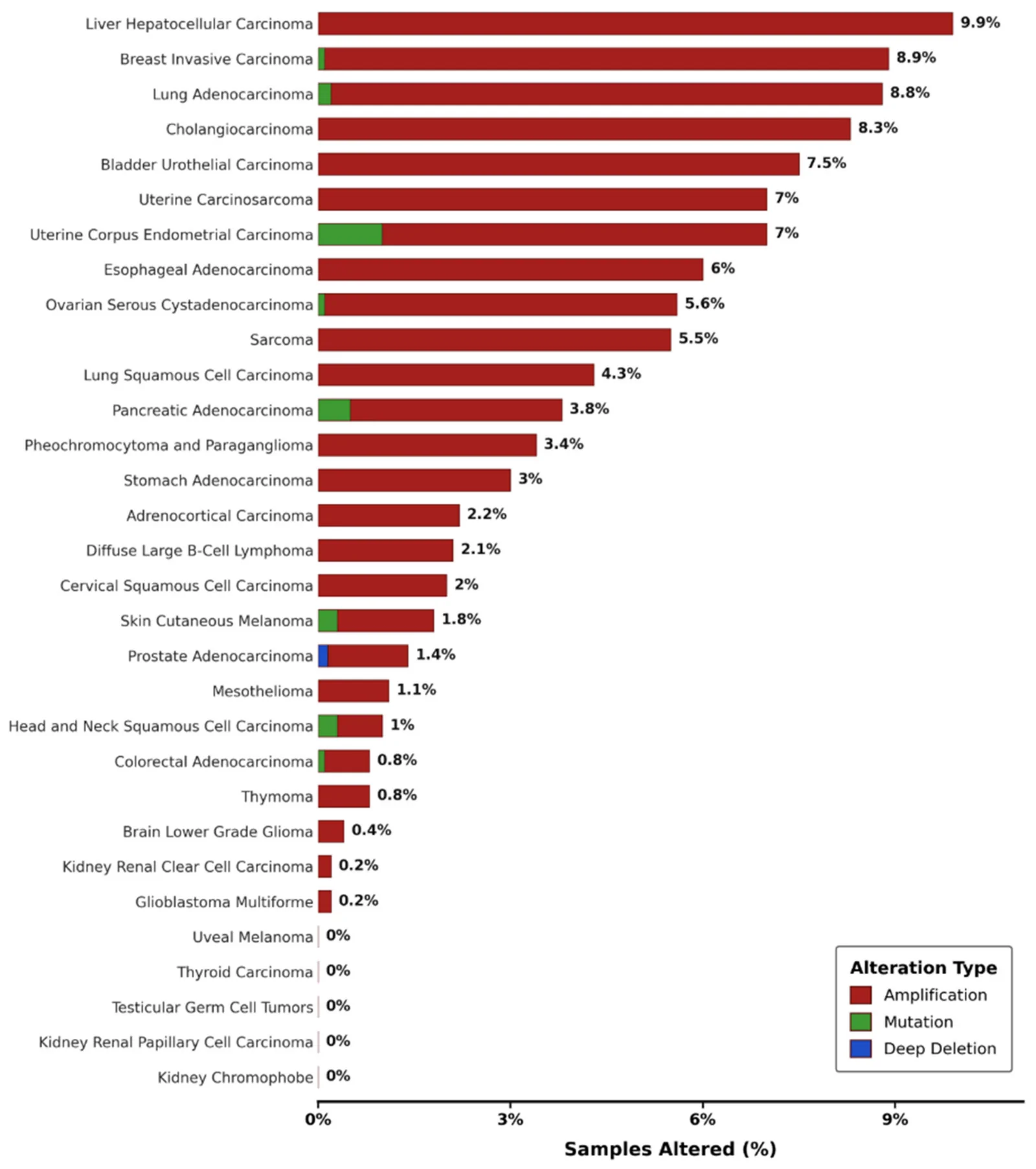
S100A11 genomic alterations across 31 TCGA PanCancer Atlas tumour types. Stacked bar plot showing the proportion of altered samples per tumour type in 10,767 tumours (cBioPortal), ordered by descending alteration frequency. Bars are colour-coded by alteration class: amplification (red), mutation (green) and deep deletion (blue). Amplification predominates across nearly all tumour types, with the highest frequencies in liver HCC (9.9%), breast invasive carcinoma (8.9%), lung adenocarcinoma (8.8%) and cholangiocarcinoma (8.3%). Mutations are proportionally most prominent in uterine corpus endometrial carcinoma, pancreatic adenocarcinoma, and head and neck squamous cell carcinoma, whereas deep deletion is essentially restricted to prostate adenocarcinoma. No alterations were detected in thyroid carcinoma, testicular germ cell tumours, uveal melanoma, or papillary and chromophobe renal carcinomas.

To systematically characterise the genomic alteration landscape of S100A11 across human malignancies, we queried the TCGA PanCancer Atlas collection on cBioPortal (31 studies, 10,767 samples). Copy number amplification was the dominant alteration class in every study with appreciable alteration frequency; non-silent mutations were rare throughout (Figure 1). Hepatocellular carcinoma (TCGA-LIHC) showed the highest S100A11 alteration frequency of any cohort in the collection (9.9%, 37/372 samples, en-tirely amplification-driven), followed by breast invasive carcinoma (8.9%), lung adeno-carcinoma (8.8%), cholangiocarcinoma (8.3%), and bladder urothelial carcinoma (7.5%). Both hepatobiliary cancers in the collection HCC and cholangiocarcinoma ranked in the top four, nominating the liver as a site of recurrent S100A11 genomic activation and providing the rationale for the focused transcriptomic investigation that follows. Gy-naecological tumours (uterine carcinosarcoma, 7%; endometrial carcinoma, 7%; ovarian serous cystadenocarcinoma, 5.6%) form a second enriched cluster, each case driven predominantly by copy number amplification. At the lower end of the spectrum, uveal melanoma, thyroid carcinoma, testicular germ cell tumours, and the two non-clear-cell renal carcinomas showed no detectable S100A11 alteration. Across this pan-cancer cohort, copy number amplification emerged as the dominant mechanism of S100A11 genomic dysregulation, vastly outnumbering somatic point mutations in nearly all cancer types investigated (Figure 1).

In contrast, somatic mutations contributed meaningfully to the alteration burden only in a subset of cancer types, most prominently uterine corpus endometrial carcinoma, ovarian serous cystadenocarinoma, pancreatic and colorectal adenocarcinoma and head and neck squamous cell carcinoma, where mutational contributions were visually appreciable alongside amplification events. Deep deletions were rare events across the cohorts and did not constitute a primary mechanism of S100A11 dysregulation in any cancer type examined. They were more visually appreciated only in prostate adenocarcinoma (Figure 1).

At the lower end of the genomic alteration spectrum, head and neck squamous cell carcinoma, colorectal adenocarcinoma, thymoma, lower-grade brain glioma, clear-cell renal carcinoma, and glioblastoma multiforme each exhibited alteration frequencies at or below 1%, consistent with tissue-specific constraints on S100A11 genomic instability. No alterations were detected in thyroid carcinoma, testicular germ cell tumours, uveal melanoma, or papillary and chromophobe renal carcinomas.

Together, these findings establish copy number amplification as the principal genomic mechanism underlying S100A11 dysregulation across human cancers, with a distinct predisposition for epithelial malignancies, and with HCC consistently occupying a place in the upper tier of alteration frequency; thus, this provides the primary rationale for deeper transcriptomic investigation in HCC.

### 3.2. Inverse Relationship Between S100A11 Copy Number Amplification and DNA Methylation Reveals Distinct Mechanisms of Transcriptional Dysregulation

**Figure 2:**
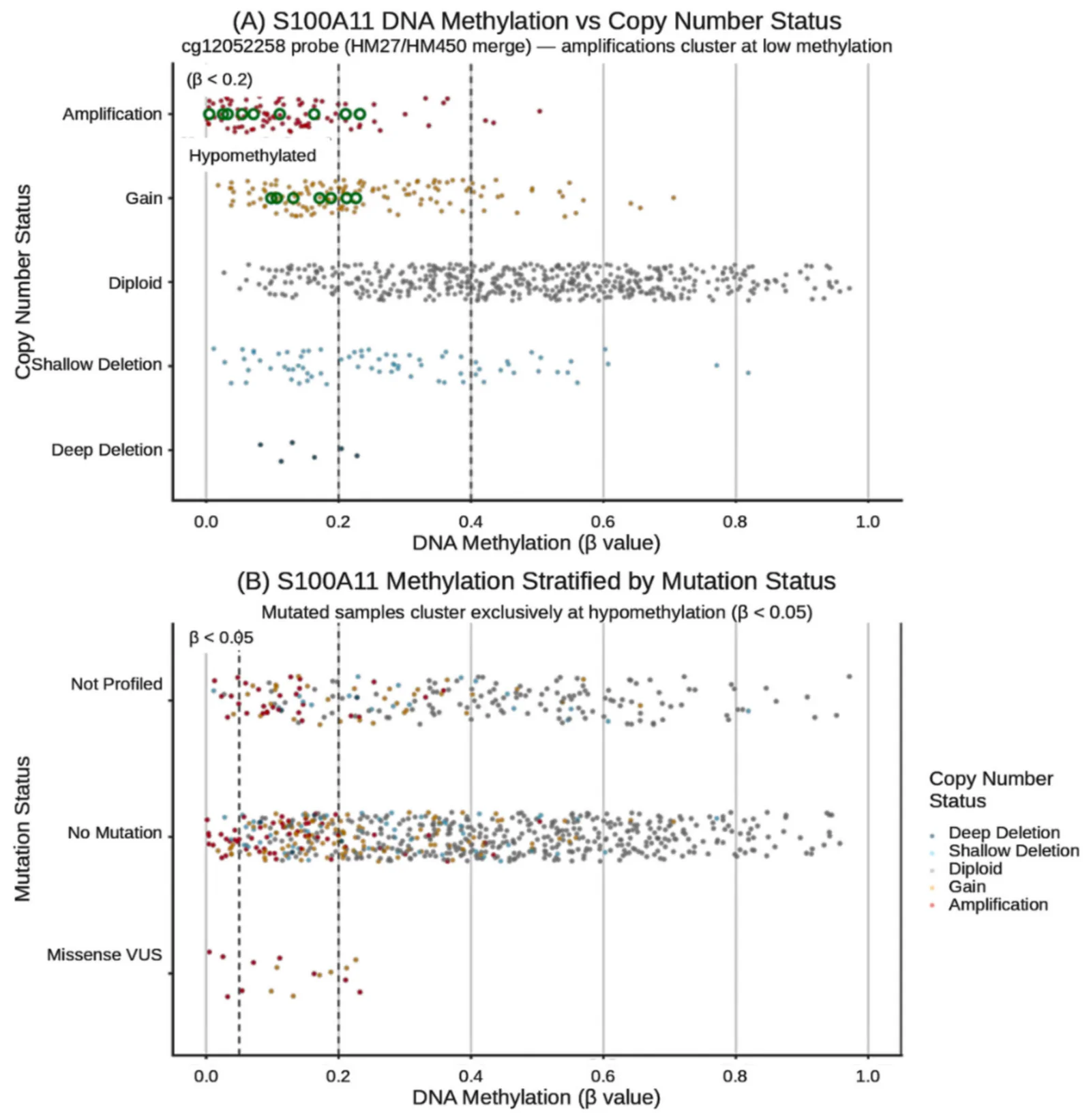
Inverse relationship between S100A11 copy number status and DNA promoter methylation across cancer samples. (**A**) Scatter plot displaying S100A11 promoter methylation levels (x-axis; cg12052258 probe, HM27/HM450 merged platform; β-value range of 0.0–1.0) stratified by copy number status (y-axis) across pan-cancer samples. Each data point represents an individual cancer sample, colour-coded by copy number alteration class: red/pink, amplification; orange/yellow, gain; grey, diploid; light blue, shallow deletion; dark blue, deep deletion. Open green circles denote missense variants of uncertain significance (VUS). Vertical dashed lines indicate methylation thresholds at β = 0.2 and β = 0.4. Amplified and gained cancers cluster predominantly within the hypomethylated range (β < 0.2), whereas diploid cancers exhibit broad methylation variability spanning the full β-range. (**B**) Scatter plot illustrating S100A11 methylation levels (x-axis; cg12052258 probe) stratified by somatic mutation status (y-axis): missense VUS (bottom), no mutation (middle), and not profiled (top). Colour coding indicates copy number status as in panel A. Missense VUS mutations occur exclusively at profound hypomethylation (β < 0.05), predominantly in diploid cancers.

Having established the pan-cancer prevalence of S100A11 copy number amplification, we next sought to determine whether epigenetic regulation, specifically promoter DNA methylation, operates coordinately or antagonistically with genomic copy number status to control S100A11 transcriptional activity. Interrogating methylation levels at the cg12052258 probe (Illumina HM27/HM450 merged platform) across tumour samples stratified by copy number state revealed a consistent inverse relationship between S100A11 copy number gain and promoter methylation (Figure 2A).

Tumours harbouring copy number amplifications clustered tightly within the hypomethylated range (β < 0.2), indicating that gene dosage gain occurs preferentially in an epigenetically permissive chromatin context. Similarly, tumours with copy number gains predominantly occupied low-to-intermediate methylation values (β < 0.2–0.4), extending the pattern and collectively suggesting that amplification-driven transcriptional activation of S100A11 is facilitated by, and possibly dependent upon, concurrent promoter hypomethylation. By contrast, diploid tumours exhibited a markedly broader methylation distribution spanning the full range of β-values (approximately 0.0–0.9), consistent with a model in which epigenetic regulation assumes the dominant role in controlling S100A11 expression where copy number remains unaltered. Shallow and deep deletions clustered at low-to-moderate methylation levels, though the biological significance of S100A11 in deep deletion cases is likely negligible given probable loss of functional alleles.

Stratification by somatic mutation status provided a complementary and reinforcing perspective (Figure 2B). Samples harbouring S100A11 missense variants of uncertain significance (VUS) clustered exclusively at profound hypomethylation levels (β < 0.05), predominantly in the diploid copy number state, indicating that somatic mutations arise specifically within transcriptionally active, unmethylated chromatin contexts capable of supporting mutant protein expression. The no-mutation group, which comprised most samples, recapitulated the full spectrum of copy number and methylation variation observed in panel A, with amplified tumours again segregating into low methylation and diploid tumours spanning the complete methylation range. Critically, missense mutations and copy number amplifications were mutually exclusive across the cohort, with no sample co-harbouring both alteration types.

### 3.3. Characterisation of the S100A11 Mutational Signatures and Clonal Architecture

**Figure 3:**
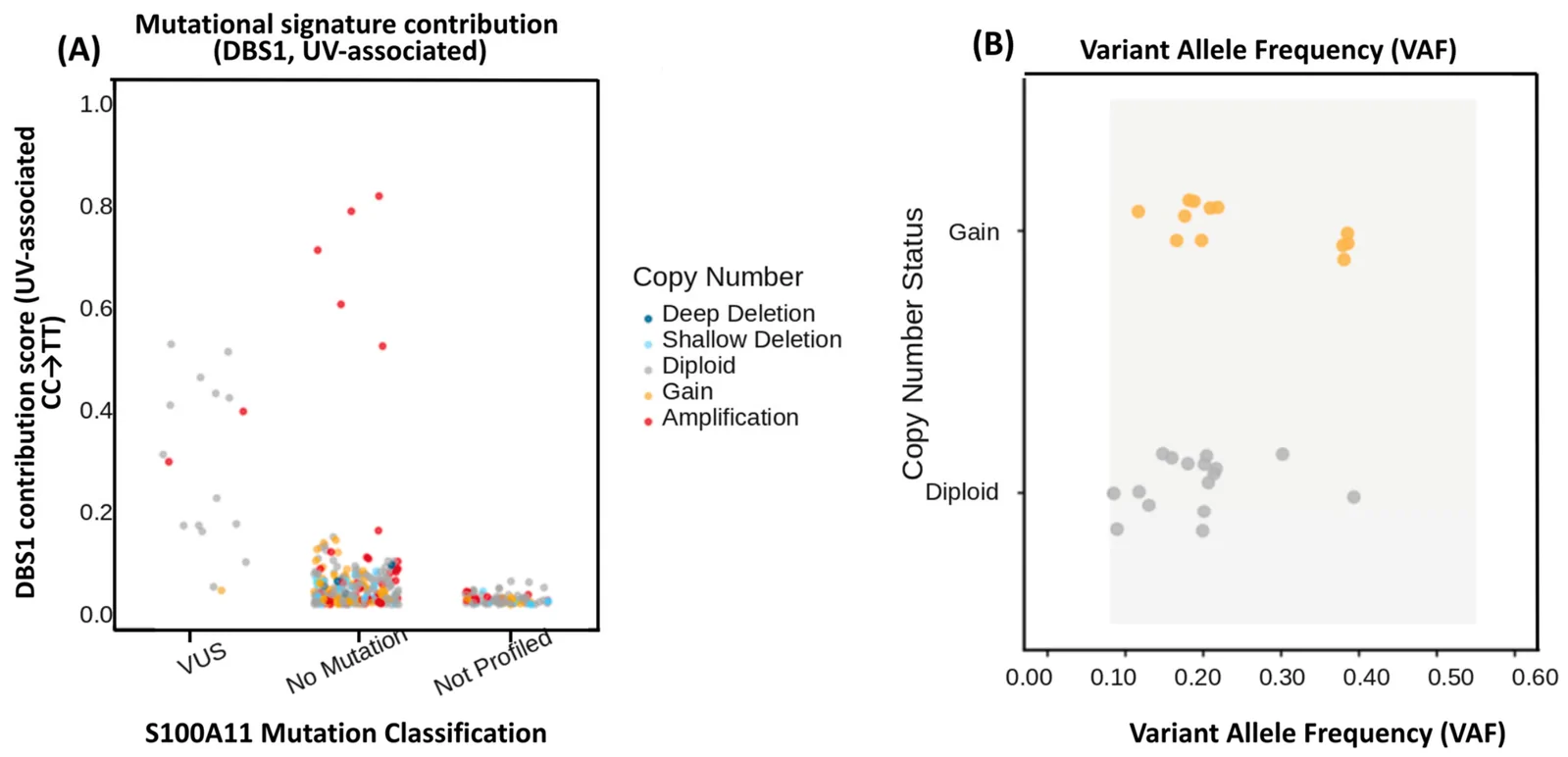
Functional characterisation of S100A11 alterations by mutational signature and clonal architecture. (**A**) Strip plot displaying DBS1 mutational signature contribution scores (UV-associated doublet base substitutions; y-axis) stratified by S100A11 mutation classification (x-axis). Each point represents an individual tumour sample, colour-coded by copy number status. Elevated DBS1 contributions in amplified samples indicate an association with UV-driven mutagenesis, consistent with the tissue origins of some affected malignancies. (**B**) Variant allele frequency (VAF) plot of S100A11 missense VUS mutations across copy number states (y-axis: gain, diploid). Each circle represents an individual mutation plotted by VAF (x-axis; range, 0.0–0.6). Orange circles denote gain samples; grey circles denote diploid samples. The shaded region indicates the clonality assessment window.

To understand the functional consequences of S100A11 genomic alterations, we integrated mutational signature analysis and variant allele frequency (VAF) data to characterise the broader molecular context in which S100A11 dysregulation operates (Figure 3). The metric utilised here in Figure 3A is COSMIC DBS1, the ultraviolet-associated doublet base substitution signature (CC > TT tandem substitutions), as reported by cBioPortal from the PCAWG signature fits of [16]; the plotted value is the fraction of each sample’s total doublet substitutions attributed to this signature and is not a measure of overall mutational burden.

Analysis of doublet base substitution mutational signature contributions (DBS1, the ultraviolet-associated CC → TT tandem substitution signature [16]) stratified by S100A11 mutation classification revealed that most non-mutated samples exhibited baseline signature contribution values at or near zero. However, a subset of S100A11-amplified cases displayed markedly elevated DBS1 contributions approaching 1.0. While DBS1 is specifically a UV-associated mutational signature, its elevated contribution in these tumours likely reflects co-occurrence with UV-driven mutagenesis, an established aetiological factor in certain cancers, including melanoma and squamous cell carcinomas. Importantly, this observation does not imply that S100A11 amplification directly causes UV-induced mutagenesis; rather, it identifies an association between S100A11 amplification and tumours bearing this mutational signature. This association may reflect tissue-specific exposure patterns or shared selective pressures in UV-exposed malignancies and underscores the context-dependent nature of S100A11 dysregulation.

VAF analysis of S100A11 missense mutations across copy number states provided insight into the clonal architecture and temporal sequence of mutational acquisition (Figure 3B). Missense VUS mutations displayed VAF values spanning 0.1–0.5, with gain-harbouring samples exhibiting a bimodal VAF distribution with clusters at 0.1–0.2 and approximately 0.35, consistent with the co-existence of early clonal and later subclonal mutational events within the same tumour type. Diploid samples harbouring missense mutations showed intermediate VAF values of approximately 0.15–0.2, with one additional subclonal event at approximately 0.3. The complete absence of VAF values exceeding 0.55 across all samples argues against homozygous mutation or loss of heterozygosity at the S100A11 locus, suggesting that retention of at least one wild-type allele confers a selective advantage.

### 3.4. S100A11 Genomic Alteration Status Does Not Independently Predict Overall Survival in Pan-Cancer TCGA Cohort

**Figure 4:**
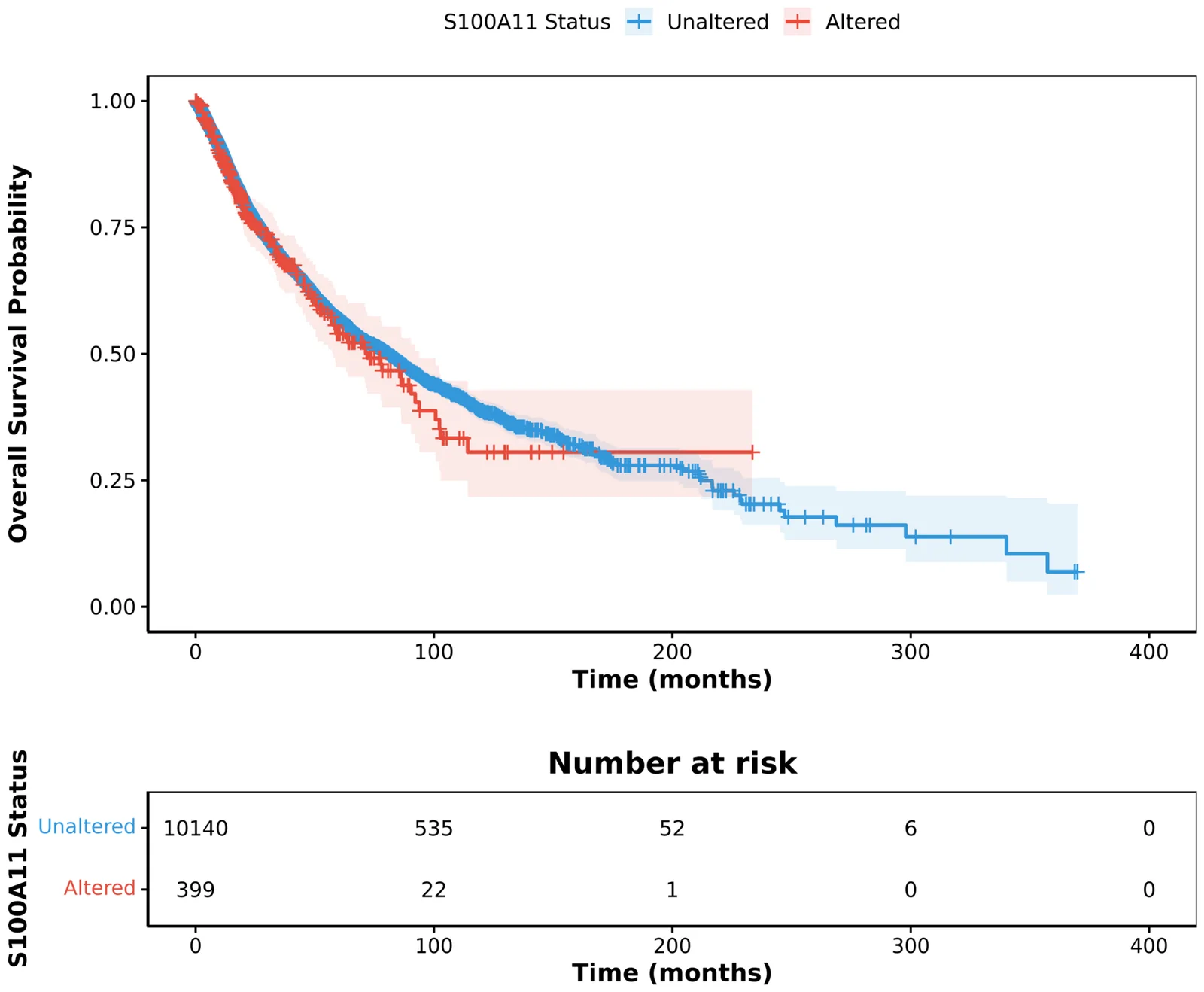
S100A11 genomic alteration status is not associated with overall survival in the TCGA PanCancer Atlas. Kaplan–Meier curves comparing overall survival in patients harbouring any S100A11 genomic alteration (amplification, deep deletion or mutation; n = 399) with unaltered patients (blue, n = 10,140) across all TCGA PanCancer Atlas cases with available overall survival data (n = 10,539). Shaded bands represent 95% confidence intervals and vertical tick marks denote censored observations. The number-at-risk in each group is tabulated below the plot. No significant difference in overall survival was observed between groups (log-rank *p* = 0.431; HR = 0.93, 95% CI 0.78–1.11). Curves are closely superimposed for the first 40 months and remain within overlapping confidence intervals throughout follow-up; apparent separation beyond 100 months reflects the small number of altered patients still at risk (n = 22 at 100 months, n = 1 at 200 months) rather than a divergence in outcome.

Having characterised the genomic and epigenetic landscape of S100A11 across cancer types, we next evaluated whether these alterations carry direct prognostic relevance. Kaplan–Meier analysis comparing patients harbouring any S100A11 genomic alteration amplification, deep deletion or mutation (n = 399) with unaltered patients (n = 10,140) across all TCGA PanCancer Atlas cases with available survival data (n = 10,539) revealed no significant difference in overall survival (log-rank *p* = 0.431; HR = 0.93, 95% CI 0.78–1.11) (Figure 4). The hazard ratio confidence interval spans unity, and the survival curves remain closely superimposed throughout the informative follow-up period. Apparent separation beyond 100 months coincides with the exhaustion of the altered cohort (n = 22 at risk at 100 months, n = 1 at 200 months) and is therefore uninterpretable. These data indicate that S100A11 alteration status alone, aggregated across histologies and alteration classes, does not stratify outcome at the pan-cancer level, an unsurprising result given that copy number gain at 1q21.3 may be functionally silent in many lineages and that amplification, deletion and mutation are unlikely to exert concordant effects.

### 3.5. S100A11 Is Markedly Overexpressed in HCC and Independently Predicts Poor Survival

**Figure 5:**
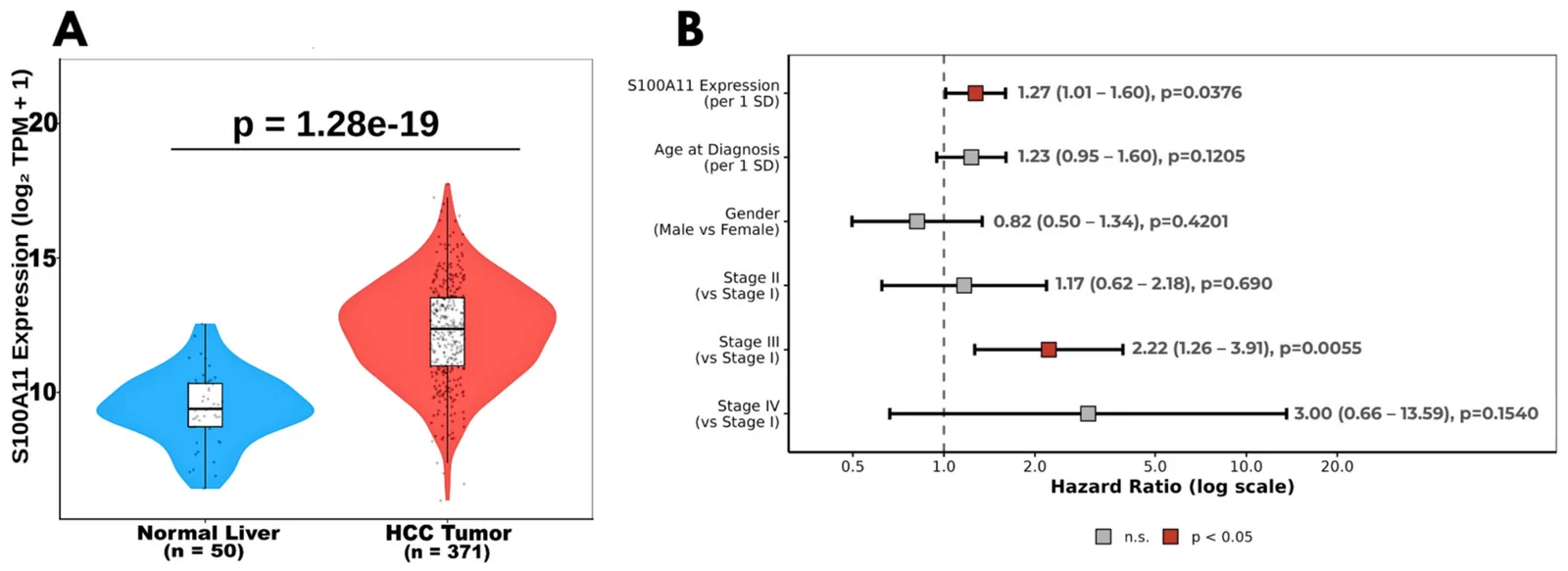
S100A11 mRNA overexpression in HCC and multivariate prognostic analysis. (**A**) Violin plots with overlaid box plots displaying S100A11 mRNA expression levels (log_2_ TPM + 1; y-axis) in normal liver tissue (blue; n = 50) and HCC tumour samples (red; n = 371) from the TCGA-LIHC cohort. Individual data points are shown with jitter overlay for visualisation. The centre line of each box plot indicates the median expression value; box boundaries represent the interquartile range. Statistical significance was assessed by Wilcoxon rank-sum test (*p* = 1.28 × 10^−19^). (**B**) Forest plot displaying hazard ratios (HRs) and 95% confidence intervals (CIs) from the multivariable Cox proportional hazards regression analysis of overall survival in TCGA-LIHC HCC patients with complete clinical and AJCC staging data (n = 239). Variables included S100A11 expression (per 1 standard deviation increase; HR = 1.27, 95% CI: 1.01–1.60), age at diagnosis, sex, and AJCC pathological stage. Stage III was independently associated with poorer overall survival (HR = 2.22, 95% CI: 1.26–3.91, *p* = 0.0055), whereas Stage II (HR = 1.17, 95% CI: 0.62–2.18, *p* = 0.629) and Stage IV (HR = 3.00, 95% CI: 0.66–13.59, *p* = 0.154) did not reach statistical significance. The vertical dashed line at HR = 1.0 represents the null effect. Hazard ratios are displayed on a log_2_ scale. Variables (age, gender, Stage II and Stage IV) are denoted as non-significant (*p* ≥ 0.05), indicated by grey squares, whilst significant variables (S100A11 expression and Stage III) are indicated by red squares.

Having established the genomic and epigenetic basis of S100A11 dysregulation, we next characterised its transcriptional output directly within HCC. Comparison of S100A11 mRNA levels between 50 normal liver samples and 371 HCC tumours (TCGA-LIHC, GDC STAR-count RNA-seq) revealed highly significant upregulation of S100A11 in tumour tissue relative to normal liver parenchyma (Wilcoxon rank-sum test, *p* = 1.28 × 10^−19^; Figure 5A).

The strength of this differential expression, spanning multiple log_2_ units at the median and achieving genome-wide significance, establishes S100A11 as a robustly overexpressed gene in HCC, providing a strong transcriptional rationale for its evaluation as a candidate diagnostic marker.

We next fitted multivariate Cox proportional hazards models to determine whether this association was independent of established clinical covariates. In the complete-case multivariable model (n = 239), which adjusted for age, sex, and AJCC pathological stage, S100A11 expression remained independently associated with overall survival (HR = 1.27, 95% CI 1.01–1.60, *p* = 0.038).

In this model, age (HR = 1.23, 95% CI 0.95–1.60, *p* = 0.121) and sex (HR = 0.82, 95% CI 0.50–1.34, *p* = 0.420) were not independently associated with survival. Stage III disease was associated with markedly worse survival relative to Stage I (HR = 2.22, 95% CI 1.26–3.91, *p* = 0.0055), whereas Stage II (HR = 1.17, 95% CI 0.62–2.18, *p* = 0.629) and Stage IV (HR = 3.00, 95% CI 0.66–13.59, *p* = 0.154) did not reach significance, the latter reflecting the small number of Stage IV cases in this cohort (n = 6) (Figure 5B).

**Figure 6:**
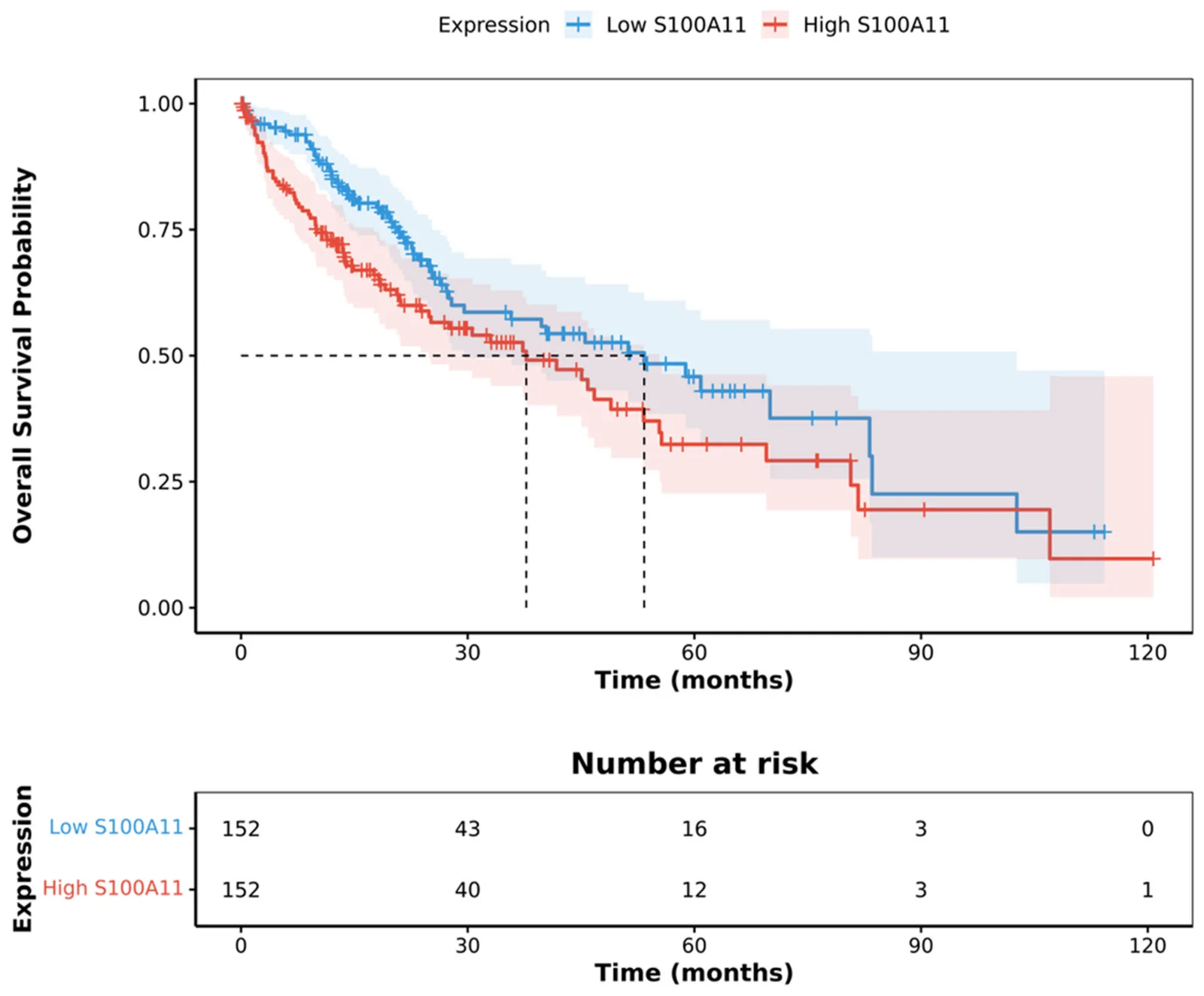
High S100A11 expression is associated with inferior overall survival in hepatocellular carcinoma. Kaplan–Meier curves comparing overall survival in TCGA-LIHC patients stratified at the median S100A11 expression value into high-expression (red, n = 152) and low-expression (blue, n = 152) groups, among 304 patients with linked expression and survival data. Shaded bands represent 95% confidence intervals and vertical ticks denote censored observations. Horizontal and vertical dashed lines indicate median overall survival in each group. Patients with high S100A11 expression showed significantly shorter overall survival than low expressers (log-rank *p* = 0.0315; HR = 1.46, 95% CI 1.03–2.06), with early curve separation evident within the first 12 months and maintained through approximately 80 months of follow-up. The number-at-risk in each group is tabulated below the plot.

We next asked whether transcriptional output, rather than genomic alteration status, carries prognostic information in HCC. Among the 304 HCC patients with linked expression and survival data, median stratification of S100A11 expression yielded balanced high (n = 152) and low (n = 152) groups. High expressers showed significantly inferior overall survival (log-rank *p* = 0.032; HR = 1.46, 95% CI 1.03–2.06), with curve separation apparent within the first 12 months and sustained through approximately 80 months of follow-up (Figure 6).

Taken together, these analyses establish a clear dissociation between genomic and transcriptional readouts of S100A11 as follows: alteration status is prognostically neutral pan-cancer, whereas elevated expression is an independent adverse prognostic factor in HCC. This dissociation is consistent with amplification representing only one of several routes to S100A11 overexpression, and motivates the transcriptional analyses presented in the following sections.

### 3.6. Pathway Enrichment Analysis Reveals S100A11-Associated Transcriptional Programmes Governing Tumour Invasion, Metabolic Reprogramming, and Immune Regulation in HCC

**Figure 7:**
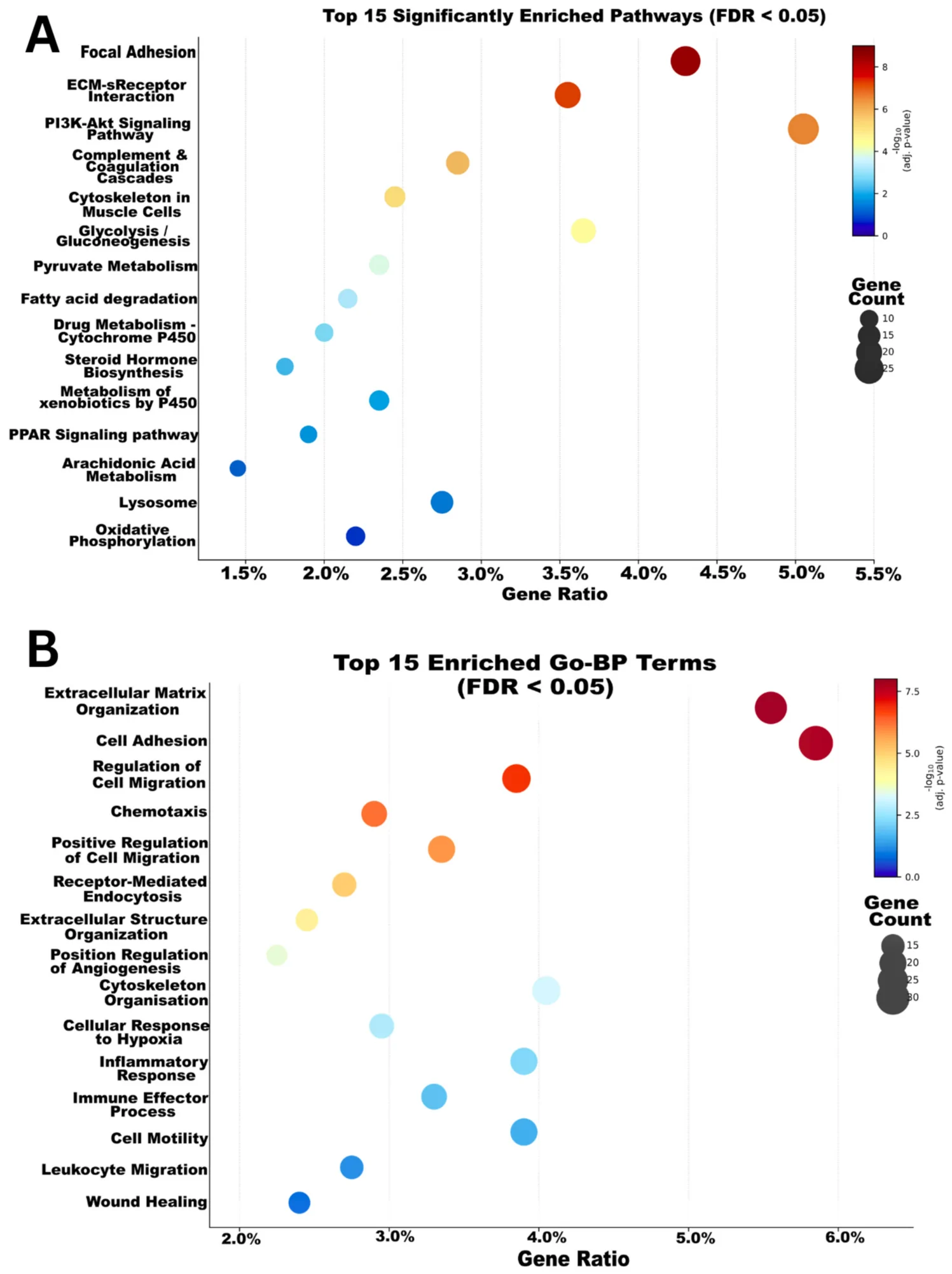
Pathway enrichment analysis in S100A11-high hepatocellular carcinoma. Dot plots displaying the top 15 significantly enriched terms (FDR < 0.05) identified from differentially expressed genes between S100A11-high and S100A11-low HCC tumours in the TCGA-LIHC cohort. The x-axis represents the gene ratio (proportion of pathway/term genes present among differentially expressed genes). Dot size is proportional to gene count and dot colour indicates statistical significance scaled by −log_10_ (adjusted *p*-value): red/dark red, highest significance; blue, lower significance. (**A**) KEGG pathway enrichment. Gene count scale: 10–25. Focal adhesion was the most significantly enriched pathway (gene ratio of ~5.5%), followed by ECM–receptor interaction and PI3K-Akt signalling. Metabolic pathways including glycolysis/gluconeogenesis, fatty acid degradation, and oxidative phosphorylation were also significantly enriched, alongside drug metabolism and PPAR signalling pathways. (**B**) Gene Ontology Biological Process enrichment. Gene count scale: 15–30. Extracellular matrix organisation and cell adhesion were the most significantly enriched terms (gene ratio of ~6%), followed by regulation of cell migration, chemotaxis, and receptor-mediated endocytosis. Immune-related terms including inflammatory response, immune effector process, and leucocyte migration were also significantly enriched, implicating S100A11 overexpression in tumour–immune microenvironment dysregulation.

To delineate the broader biological programmes associated with S100A11 transcriptional upregulation in HCC, we performed KEGG pathway and Gene Ontology Biological Process (GO-BP) enrichment analyses on differentially expressed genes between S100A11-high and S100A11-low tumour groups. KEGG pathway analysis identified focal adhesion as the most significantly enriched pathway in S100A11-high tumours (gene ratio of ~5.5%, FDR < 0.05), followed by ECM–receptor interaction and PI3K–Akt signalling (Figure 7A).

Notably, multiple metabolic pathways were significantly enriched, including glycolysis/gluconeogenesis, pyruvate metabolism, fatty acid degradation, and oxidative phosphorylation, collectively indicating that S100A11-high HCC tumours undergo extensive metabolic reprogramming. The co-enrichment of drug metabolism pathways including cytochrome P450-mediated xenobiotic metabolism, steroid hormone biosynthesis, and PPAR signalling further suggests that elevated S100A11 expression may be associated with altered drug biotransformation capacity, with implications for therapeutic response and potentially resistance in HCC.

GO-BP enrichment analysis provided complementary and orthogonal biological resolution, with extracellular matrix organisation and cell adhesion emerging as the most significantly enriched terms (gene ratio of ~6%, FDR < 0.05), closely followed by regulation of cell migration and chemotaxis (Figure 7B). Importantly, immune-related GO-BP terms including inflammatory response, immune effector process, and leucocyte migration were significantly enriched in S100A11-high tumours, suggesting that elevated S100A11 expression is coupled to immune microenvironment dysregulation and may influence the balance between pro- and anti-tumour–immune activity within the HCC tumour milieu. Additional enrichment of wound healing, cellular response to hypoxia, and positive regulation of angiogenesis collectively indicate that S100A11-high HCC occupies a transcriptionally aggressive state characterised by the simultaneous activation of invasion, immune evasion, and adaptive stress response programmes.

### 3.7. Co-Expression Network Analysis Identifies Two S100A11-Associated Transcriptional Modules in HCC

**Figure 8:**
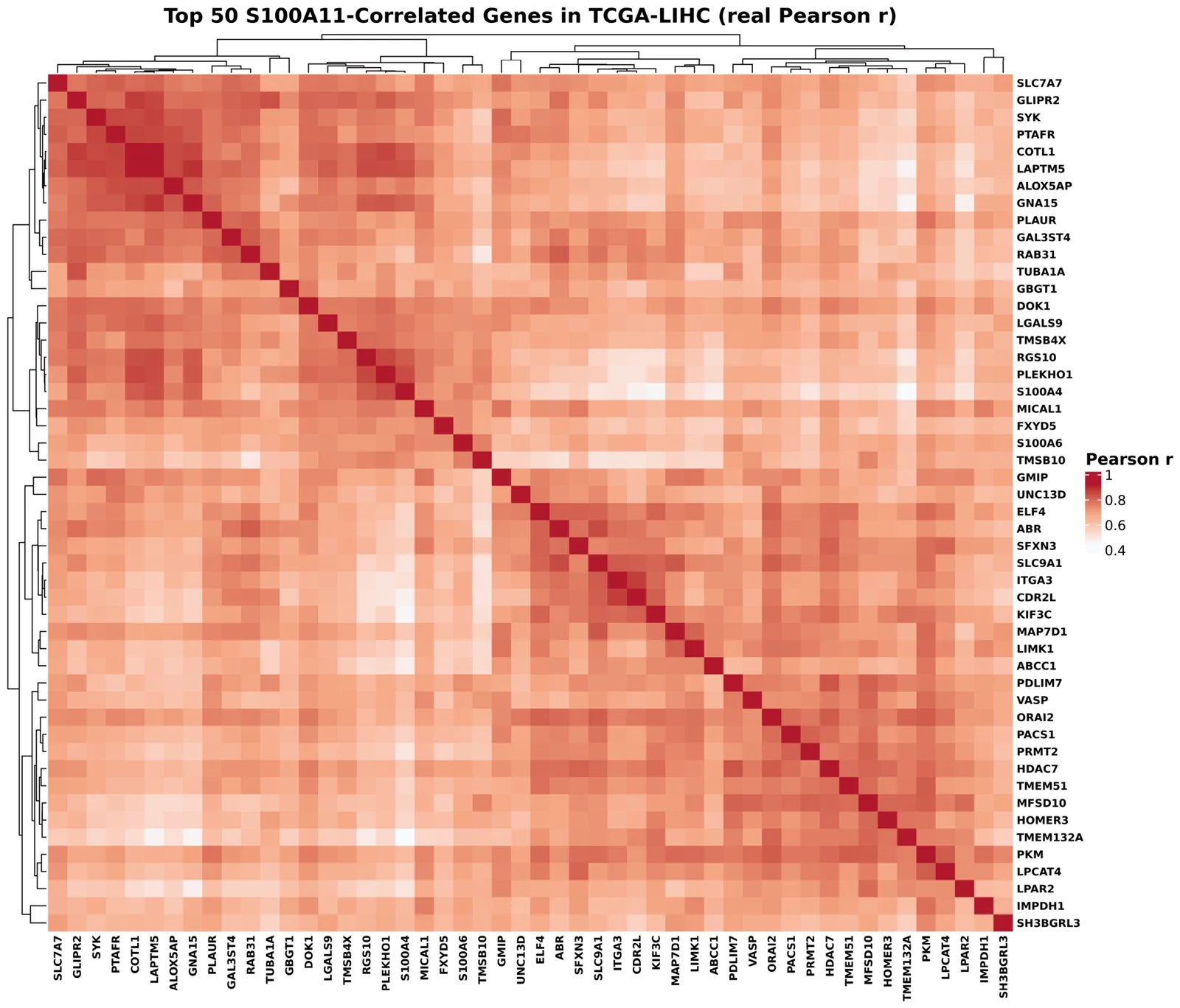
Co-expression correlation heatmap of the top 50 S100A11-correlated genes in hepatocellular carcinoma. Symmetric Pearson correlation matrix showing pairwise coefficients among the 50 genes most strongly co-expressed with S100A11 across TCGA-LIHC tumour samples. Rows and columns are ordered by hierarchical clustering (dendrograms shown on the left and top margins), which resolves two co-expression modules. The colour scale represents the Pearson correlation coefficient, ranging from approximately 0.4 (white) to 1.0 (deep red); the dark diagonal denotes self-correlation (r = 1). All pairwise correlations within this gene set are positive. Colour scale is sequential white-to-red scale (ComplexHeatmap colorRamp2) and stretched to the real off-diagonal correlation range (r ≈ 0.44–0.92), not a fixed −1 to 1 diverging scale, as all pairwise correlations among the displayed genes are positive. The first module (immune–cytoskeletal) is dominated by innate immune and myeloid-associated transcripts, including SYK, LAPTM5, ALOX5AP, PTAFR, COTL1, GNA15, DOK1, LGALS9 and RGS10, together with cytoskeletal and motility regulators (TMSB4X, TMSB10, PLEKHO1, MICAL1) and the S100 family members S100A4 and S100A6. The second module (tumour-intrinsic metabolic–adhesion) comprises genes associated with adhesion and cytoskeletal remodelling (ITGA3, LIMK1, VASP, PDLIM7), membrane transport and drug efflux (SLC9A1, ABCC1, MFSD10), transcriptional and epigenetic regulation (ELF4, HDAC7, PRMT2), and metabolic reprogramming (PKM, IMPDH1, LPCAT4, SFXN3). Inter-module correlations, although positive, are attenuated relative to intra-module correlations, indicating partial rather than complete transcriptional co-regulation between the two programmes.

To delineate the co-expression network of S100A11 in HCC, we computed Pearson correlation coefficients between S100A11 and all expressed genes across TCGA-LIHC primary tumour samples (n = 371). This identified 1237 significantly correlated genes (|r| > 0.5, *p* < 0.001), of which the 50 most strongly correlated were selected for hierarchical clustering and visualisation (Figure 8). The strongest correlates included the S100 family members S100A6 (r = 0.82) and S100A4 (r = 0.71), the glycolytic enzyme PKM (r = 0.79), the invasion-associated urokinase receptor PLAUR (r = 0.76), and the ITAM-proximal kinase SYK (r = 0.72) alongside ALOX5AP, LAPTM5, UNC13D, GNA15 and PTAFR, a coherent innate immune/myeloid signalling cluster. It also included the cytoskeletal regulators TMSB10, TMSB4X and VASP. All pairwise correlations within this gene set were positive (r ≈ 0.4–1.0), indicating a coherently co-activated transcriptional neighbourhood rather than reciprocally regulated programmes. The significant co-expression network (1237 genes in total, |r| > 0.5, *p* < 0.001) additionally included TGFB1 (r = 0.67), VIM (r = 0.68), and the ITAM-adaptor/myeloid markers ADGRE5 (r = 0.67), TYROBP (r = 0.65), MRC2 (r = 0.64), FCER1G (r = 0.62), HLA-DMB (r = 0.61), CYBB (r = 0.60), ITGB2 (r = 0.60), and COL11A1 (r = 0.53) which are all significant, though ranked just below the top 50 cutoff used for the heatmap visualisation.

Hierarchical clustering resolved this gene set into two co-expression modules with distinct biological identities (Figure 8). The first module was dominated by innate immune and myeloid-associated transcripts, including SYK, LAPTM5, ALOX5AP, PTAFR, COTL1, GNA15, DOK1, LGALS9 and RGS10, which co-clustered tightly with cytoskeletal and motility regulators (TMSB4X, TMSB10, PLEKHO1, MICAL1) and the S100 family members S100A4 and S100A6. The composition of this module suggests that S100A11-high tumours are characterised by preferential myeloid infiltration coupled to enhanced cytoskeletal and migratory programmes, a pattern consistent with an immunosuppressive rather than cytotoxic tumour microenvironment.

The second module comprised predominantly tumour-intrinsic genes governing adhesion and cytoskeletal remodelling (ITGA3, LIMK1, VASP, PDLIM7), membrane transport and drug efflux (SLC9A1, ABCC1, MFSD10, ORAI2), transcriptional and epigenetic regulation (ELF4, HDAC7, PRMT2), and metabolic reprogramming (PKM, IMPDH1, LPCAT4, SFXN3). The presence of PKM and IMPDH1 links elevated S100A11 expression to glycolytic and nucleotide-biosynthetic reprogramming, while ABCC1 co-expression raises the possibility of an association with multidrug-resistant phenotypes.

Inter-module correlations, although uniformly positive, were attenuated relative to intra-module correlations, as reflected in the lighter off-diagonal regions of the matrix (Figure 8). This indicates partial rather than complete transcriptional co-regulation between the immune–cytoskeletal and tumour-intrinsic metabolic programmes, suggesting that elevated S100A11 expression is associated with two related but separably regulated transcriptional axes. Together, these findings support a model in which high S100A11 marks an aggressive HCC molecular state defined by the coordinated activation of invasion, metabolic reprogramming and innate immune remodelling.

**Figure 9:**
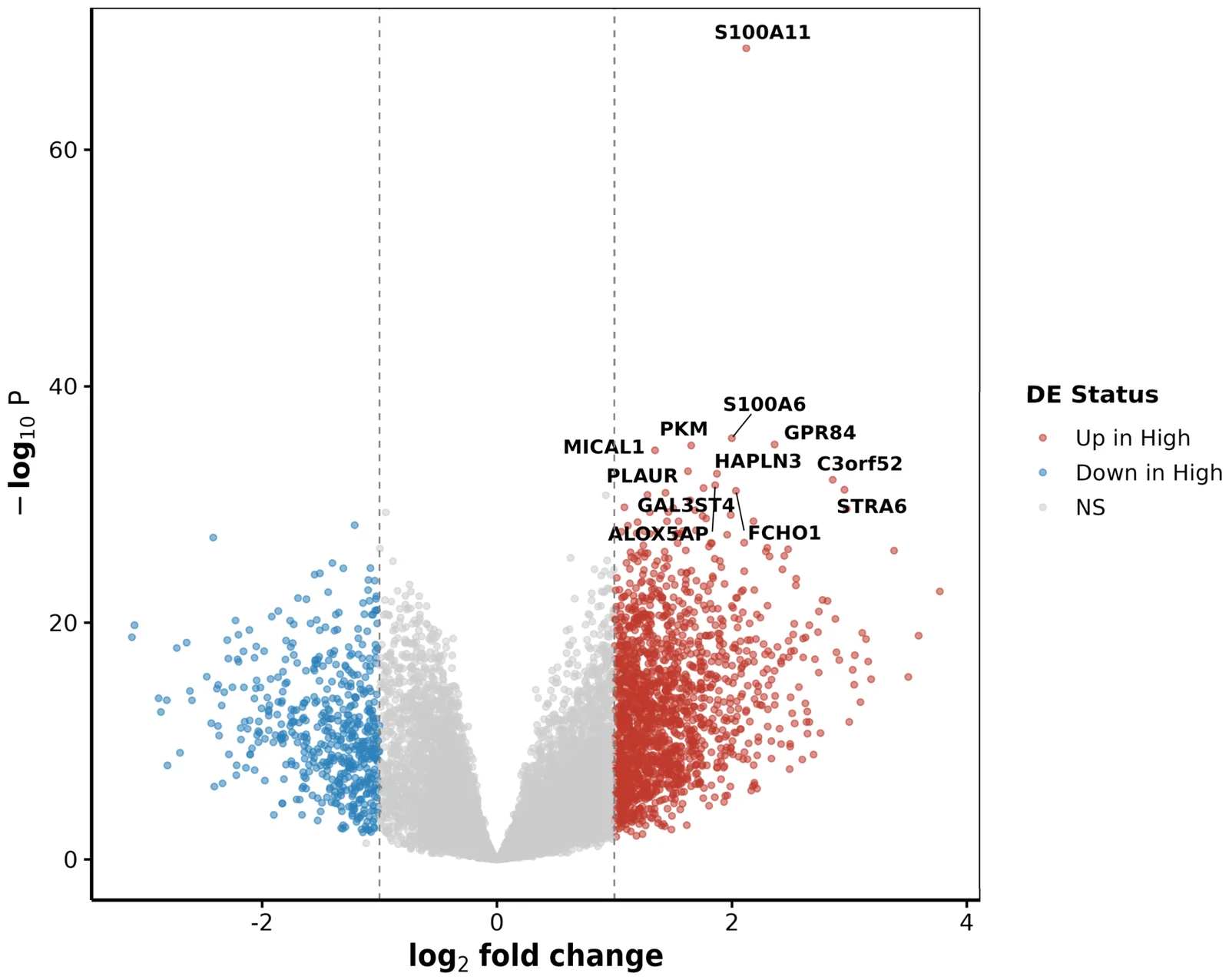
Differential expression between S100A11-high and S100A11-low HCC tumours (TCGA-LIHC). Volcano plot of limma-voom differential expression analysis comparing median-stratified S100A11-high (n = 186) and S100A11-low (n = 185) primary tumours. Points represent individual genes plotted by log_2_ fold change against −log_10_P; dashed lines mark |log_2_FC| = 1. Red, upregulated in S100A11-high; blue, downregulated; grey, non-significant. A total of 2194 genes were differentially expressed (FDR < 0.05, |log_2_FC| > 1). The twelve most significant upregulated genes are labelled. DE: differentially expressed, NS: not significant.

Median stratification of the 371 TCGA-LIHC primary tumours yielded balanced S100A11-high (n = 186) and S100A11-low (n = 185) groups, from which 2194 genes emerged as differentially expressed. The distribution is strongly asymmetric: upregulated genes substantially outnumber downregulated genes and extend to larger effect sizes (log_2_ FC up to ~3.8 versus ~−3.1), indicating that elevated S100A11 expression is associated predominantly with transcriptional activation rather than repression. S100A11 itself is the most significant hit (−log_10_P ≈ 70) (Figure 9).

The labelled genes fall into three coherent functional groups that reinforce the co-expression modules described in Figure 8. Firstly, the metabolic reprogramming that includes PKM, encoding pyruvate kinase M2, together with STRA6 (retinol transport) and GAL3ST4 (glycosphingolipid sulfation), is consistent with glycolytic and lipid metabolic rewiring in S100A11-high tumours. Invasion and cytoskeletal remodelling represent the PLAUR, MICAL1, HAPLN3 and FCHO1, implicating urokinase-mediated pericellular proteolysis, actin regulation and hyaluronan-linked matrix organisation. Innate immune and myeloid signalling reflect ALOX5AP and GPR84, both restricted largely to myeloid lineages, alongside S100A6, supporting the myeloid-enriched microenvironment inferred from the upper co-expression module. The convergence of PKM, S100A6, PLAUR, MICAL1, ALOX5AP and GAL3ST4 across both the correlation-based (Figure 8) and stratification-based (Figure 9) analyses provides methodological cross-validation, since these are statistically independent approaches to the same underlying transcriptomic data.

## 4. Discussion

This study provides a multi-layered characterisation of S100A11 dysregulation across human malignancies and establishes its context-dependent functional and clinical relevance, with particular significance in hepatocellular carcinoma (HCC). By integrating pan-cancer genomics, epigenetic profiling, and HCC-focused transcriptomics and co-expression network analyses, we demonstrate that S100A11 operates within a complex regulatory landscape in which genomic and epigenetic mechanisms converge to shape tumour behaviour. These findings are consistent with recent pan-cancer analyses identifying S100A11 as a broadly upregulated oncogenic factor associated with tumour progression and immune modulation [17].

A central finding of the present study is that copy number amplification represents the dominant mechanism of S100A11 genomic dysregulation across cancers. Previous studies have primarily characterised S100A11 through its transcriptional overexpression in individual tumour types, including pancreatic, colorectal, and HCC [5,6,7,8]. Our results extend these observations by demonstrating that this overexpression is frequently underpinned by genomic amplification and exhibits strong tissue specificity, with the highest frequencies observed in epithelial malignancies including HCC and lung adenocarcinoma. This is consistent with pan-cancer analyses of the broader S100 protein family, which have demonstrated lineage-dependent expression patterns and associations with immune infiltration [18]. These findings also align with earlier work in the Karpas-299 anaplastic large cell lymphoma cell line, in which elevated S100A11 mRNA was attributed to DNA amplification [19].

We further identify a robust inverse relationship between S100A11 copy number amplification and promoter methylation, indicating that transcriptional activation requires an epigenetically permissive chromatin state, consistent with models of oncogene activation in which promoter hypomethylation cooperates with genomic amplification to sustain transcriptional upregulation [20,21]. The frequency of somatic mutations was very low compared with amplification, consistent with reported S100A11 mutation rates of approximately 5% in low-grade glioma [22] and across pan-cancer datasets [17]. Strikingly, amplification and somatic mutation were mutually exclusive across the entire cohort, defining two molecularly distinct routes to S100A11 dysregulation: amplification-driven overexpression in hypomethylated contexts, and mutation-driven alteration in transcriptionally active diploid tumours, each with distinct implications for patient stratification and therapeutic targeting. This mutual exclusivity reflects a broader principle in cancer genomics whereby distinct alteration types converge on the same oncogenic pathway without co-occurring, as exemplified by mutual exclusivity in PI3K, p53, and Rb pathway alterations in glioblastoma, and between BRCA1/2 inactivation and CCNE1 amplification in serous ovarian cancer [23].

From a clinical perspective, our findings reveal an important dissociation between genomic and transcriptional readouts of S100A11 in HCC. Genomic alteration status was prognostically neutral at the pan-cancer level, with no statistically significant survival difference between altered and unaltered patients. The wide confidence interval and limited size of the altered cohort preclude meaningful inference regarding a survival effect attributable to genomic alteration status alone. In contrast, transcriptional upregulation of S100A11 in HCC emerges as prognostically significant, with high expression associated with significantly inferior overall survival, in addition to markedly elevated expression in tumour relative to normal liver tissues. This pattern is consistent with recent studies demonstrating that elevated S100A11 expression correlates with tumour aggressiveness and adverse clinical features across multiple cancer types. For example, S100A11 expression was associated with poor survival in breast cancer [24] and identified as a serum biomarker in non-metastatic pancreatic cancer [25], and its protein expression as an unfavourable prognostic indicator in surgically resected pancreatic adenocarcinoma [26]. The strong discriminatory capacity of S100A11 expression in distinguishing HCC from normal liver tissue supports its candidacy as a diagnostic marker, warranting prospective validation, which is consistent with prior reports of a diagnostic role for S100A11 in HCC [8,14,27,28].

A relevant question is how S100A11 might compare with other candidate biomarkers, including the related family member S100A10, which has also been implicated in liver disease and HCC. Recent functional work using complementary mouse models of MASLD-driven hepatocarcinogenesis found that these two closely related S100 family members play opposing roles in liver tumour development: hepatocyte-specific knockdown of S100A11 consistently reduced tumour growth, confirming an oncogenic function, whereas knockdown of S100A10 unexpectedly promoted hepatocarcinogenesis, and its overexpression instead reduced tumour volume, identifying S100A10 as tumour-suppressive rather than oncogenic in this context [15]. This functional divergence has direct implications for biomarker selection: whereas elevated S100A10 expression would be difficult to interpret as a straightforward marker of tumour aggressiveness given its protective role, the oncogenic, pro-tumorigenic function demonstrated for S100A11 is directly concordant with the diagnostic and prognostic associations identified in the present study, including its overexpression in tumour relative to normal liver tissue, its independent adverse prognostic value, and its positive correlation with Stage III disease. This concordance between functional role and clinical association strengthens the biological plausibility of S100A11 as a marker of aggressive HCC biology.

Mechanistically, pathway enrichment analyses converge on a model in which S100A11 overexpression is associated with coordinated activation of ECM remodelling, cytoskeletal reorganisation, metabolic reprogramming, and immune modulation. The prominence of focal adhesion, ECM–receptor interaction, and PI3K–Akt signalling among the enriched pathways is consistent with experimental evidence linking S100A11 to invasion and metastasis in HCC through AKT and ERK signalling [8]. Emerging evidence implicates intracellular S100A11 in additional oncogenic mechanisms including desmosome-associated signalling, β-catenin/TCF activation and focal adhesion [4,29]. The co-occurring enrichment of wound healing, hypoxia response, and angiogenesis programmes suggests that S100A11-high HCC occupies a transcriptionally aggressive state defined by the simultaneous activation of invasion, immune evasion, and adaptive stress response.

The enrichment of metabolic pathways, including glycolysis, pyruvate metabolism, fatty acid degradation, and oxidative phosphorylation, suggests S100A11 contributes to the metabolic plasticity central to tumour adaptation and therapeutic resistance [30]. HCC is characterised by extensive metabolic reprogramming encompassing glycolysis, the pentose phosphate pathway, amino acid metabolism, and fatty acid metabolism [31], and the sequential shift from gluconeogenesis and glycogen storage to aerobic glycolysis is a recognised early event in hepatocarcinogenesis [32]. The co-expression of glycolytic and nucleotide-biosynthetic genes alongside S100A11 lends further support to this link and is consistent with reports of insulin receptor pathway upregulation as a potential early regulator of the same metabolic shift [33]; the precise contribution of S100A11 to metabolic reprogramming in HCC warrants dedicated investigation.

The co-enrichment of cytochrome P450-mediated drug metabolism and PPAR signalling pathways raises further hypotheses regarding therapeutic resistance. Sorafenib, the first-line agent for advanced HCC, is metabolised primarily through CYP3A4-mediated oxidation, and hepatic CYP activity is a recognised determinant of pharmacokinetics and treatment response [34,35]. Sorafenib metabolism is significantly altered in HCC tumour tissue relative to normal liver, with reduced CYP expression potentially contributing to therapeutic failure [36]. The enrichment of these pathways in S100A11-high tumours, together with the co-expression of a known multidrug-resistant transporter, raise the possibility that elevated S100A11 is associated with dysregulated drug biotransformation and efflux, thus warranting functional validation.

The enrichment of immune-related pathways is consistent with an active role for S100A11 in shaping the tumour–immune microenvironment. Cancer-cell-derived S100A11 promotes monocyte recruitment and macrophage infiltration in a dose-dependent manner in ER+ breast cancer models, with S100A11 silencing reducing monocyte infiltration and expression of the immunosuppressive marker CD206, establishing S100A11 as a paracrine regulator of cancer–macrophage crosstalk [37]. Consistent with this, S100A11 expression correlates with M2 macrophage polarisation and an immunosuppressive microenvironment in glioma [38], and single-cell RNA sequencing data in HCC implicate S100A11 in regulating tumour-infiltrating immune cells, particularly T-cell function via NF-κB signalling [39]. In agreement with the literature, our co-expression network demonstrated that the immune-associated module is anchored by innate immune and myeloid-signalling transcript consistent with a preferentially immunosuppressive rather than cytotoxic tumour microenvironment mediated through myeloid ITAM-coupled activation pathways. TYROBP, FCER1G, and ADGRE5 are real, significant S100A11 correlates in our analysis, placing within the broader real co-expression network alongside the top 50 myeloid signalling genes SYK, ALOX5AP, LAPTM5, and PTAFR shown in Figure 8. Pan-cancer analyses have similarly linked S100A11 to immune checkpoint regulation and immunosuppressive cell infiltration [17], collectively positioning S100A11 as a mediator of tumour–immune crosstalk with implications for immunotherapy response in HCC.

The co-expression network identifies two partially independent transcriptional modules linking S100A11 with metabolic, cytoskeletal, and immune regulatory programmes. Co-expression with S100A4 and S100A6 is consistent with systems-level analyses of S100 protein function in cancer [1]. Convergence between the correlation-based and differential-expression-based analyses around a shared set of invasion, glycolytic, and myeloid markers provides methodological cross-validation, as these represent statistically independent approaches applied to the same underlying transcriptomic data. The biological programmes represented across both modules, ECM remodelling, cytoskeletal reorganisation, metabolic reprogramming, and immune dysregulation map plausibly onto established HCC transcriptomic subclasses, including the proliferative and immune-active subtypes [13], a correspondence warranting direct investigation in subtype-annotated cohorts.

The present study has the following limitations: Reliance on retrospective, publicly available data introduces potential cohort biases, and the relatively small number of S100A11-altered cases limits statistical power for survival analyses based on genomic alteration status. The multivariate Cox regression, whilst directionally consistent with established HCC epidemiology, was similarly underpowered in the subset of patients with complete clinical data. Although S100A11 expression showed a positive correlation with Stage III disease, supporting a link between elevated expression and more advanced tumour stage, the cross-sectional nature of this association means it cannot establish whether S100A11 upregulation precedes or follows the transition to Stage III disease, and the prognostic association reported here should therefore continue to be interpreted as correlative. Furthermore, whilst our integrative analyses generate strong mechanistic hypotheses regarding the pathways linking S100A11 to invasion, metabolic reprogramming, and immune modulation, experimental validation is required to establish causality, particularly regarding the interplay between genomic amplification, epigenetic remodelling, and downstream signalling. The absence of protein-level validation represents a further limitation, as mRNA expression does not necessarily reflect protein abundance or activity. Finally, all analyses were conducted in a single TCGA cohort; independent replication in prospective or externally validated datasets is essential before any clinical translation of these findings can be considered.

## 5. Conclusions

This study provides an integrated genomic and epigenetic characterisation of S100A11 dysregulation across human cancers, with a focused transcriptomic investigation in HCC. Copy number amplification, operating in concert with promoter hypomethylation, is the dominant mechanism driving S100A11 overexpression across epithelial malignancies, whilst the mutual exclusivity of amplification and somatic mutation defines two mechanistically distinct routes to dysregulation, placing S100A11 within a broader model of epigenetically permissive oncogene activation.

S100A11 overexpression was found to be associated with the coordinated activation of invasion, metabolic reprogramming, and immune evasion programmes. Unlike genomic alteration status, which is prognostically neutral at the pan-cancer level, elevated S100A11 transcriptional expression is an independent adverse prognostic factor in HCC and correlates positively with Stage III disease, together supporting a role for S100A11 in more advanced, invasive tumour biology. Its strong discriminatory capacity between HCC and normal liver tissue further supports its candidacy as a diagnostic and prognostic marker in established disease, whilst its transcriptional network, spanning invasion, metabolic, and immune programmes, identifies potential vulnerabilities for therapeutic intervention.

Future studies should prioritise protein-level validation in independent prospective cohorts, along with functional studies in HCC models to establish causality, preclinical and clinical testing of the association between S100A11 and resistance to sorafenib. Additionally, replication studies in multi-centre subtype-annotated cohorts should be conducted before clinical translation is considered. The emerging role of S100A11 in focal adhesion and metabolic reprogramming should be investigated during the transition from preneoplastic to neoplastic phenotype in HCC.

## Data Availability

All data analysed in this study are derived from publicly available repositories. S100A11 genomic alteration data were obtained from cBioPortal (https://www.cbioportal.org/, [accessed on 10 December 2025]), encompassing 31 studies and 10,767 samples sourced from The Cancer Genome Atlas (TCGA; https://www.cancer.gov/ccg/research/genome-sequencing/tcga, [accessed on 10 December 2025]). DNA methylation and mutational signature were accessed via RNA sequencing data and clinical information for the HCC-specific analyses were obtained from the TCGA Liver Hepatocellular Carcinoma (TCGA-LIHC) project through the Genomic Data Commons (GDC) Data Portal (https://portal.gdc.cancer.gov/, [accessed on 10 December 2025]).
